# A New Pathologic *KMT2B* Variant Associated with Childhood Onset Dystonia Presenting as Variable Phenotypes among Family Members

**DOI:** 10.5334/tohm.679

**Published:** 2022-03-17

**Authors:** Laura R. Owczarzak, Kelsey E. Hogan, Richard T. Dineen, Chandler E. Gill, Mindy H. Li

**Affiliations:** 1 Rush Medical College and Rush University Medical Center, Chicago, IL, USA; 2 Division of Genetics, Department of Pediatrics, Rush Medical College and Rush University Medical Center, Chicago, IL, USA; 3 Section of Movement Disorders, Department of Neurological Sciences, Rush University Medical Center, Chicago, IL, USA

**Keywords:** KMT2B, dystonia, DYT-28, variable expressivity, MLL4, complex dystonia

## Abstract

**Background:**

*KMT2B*-related dystonia is a primarily childhood-onset movement disorder characterized by progressive dystonia, spasticity, and developmental delay. A minority of individuals possess an inherited *KMT2B* variant.

**Case Report:**

As a child, the proband experienced mild developmental delay and laryngeal dystonia which progressed to generalized dystonia. Patellar hyperreflexia, postural tremor, and everted gait were documented. Whole exome sequencing identified a heterozygous pathogenic *KMT2B* variant in the proband, proband’s sister, and proband’s mother who had milder presentations.

**Discussion:**

This novel *KMT2B* variant reflects intrafamilial variable expressivity in *KMT2B*-related dystonia. Further identification of variants will allow for better appreciation of the phenotypic spectrum.

## Background

*KMT2B*-related dystonia is a rare complex movement disorder characterized by variants in *KMT2B*. *KMT2B* (also known as *MLL4*) codes for a histone lysine methyl-transferase involved in the methylation of histone H3 and lysine 4 (H3K4) and epigenetic modification associated with active gene transcription [1]. KMT2B is expressed ubiquitously throughout human tissues, with reduced expression in *KMT2B-*related dystonia patients [2]. Notably, KMT2B expression in the brain is greatest within the cerebellum.

*KMT2B*-related dystonia is an autosomal dominant disease which presents primarily in childhood with lower extremity dystonia, gait disturbance, and abnormal foot posturing [4,5,6,7]. A significant portion of individuals also initially present with upper limb dystonia. *KMT2B*-related dystonia may progress into generalized dystonia of limbs, larynx, and face. This can lead to dysphagia and dysphonia, occurring on average two to eleven years after initial symptom onset. Patients may also develop spasticity. Reported severity varies, ranging from severe dystonia necessitating wheelchair confinement to mild cognitive impairment and minor gait disturbance. Other associated clinical findings include psychiatric disorders (such as attention-deficit/hyperactivity disorder, obsessive-compulsive disorder, and depression), developmental delay, sensorineural hearing loss, myoclonus, and seizures [2,3].

To date, less than eighty affected individuals have been identified with a wide variety of genotypes [3]. Diagnosis can be made via chromosomal microarray, targeted dystonia panel or whole exome sequencing [2,4,5,6,14]. Testing may identify a heterozygous pathogenic variant of *KMT2B* or heterozygous deletion of 19q13.12 that includes *KMT2B*. To date, the majority (84%) of identified individuals with *KMT2B*-related dystonia have a de novo pathogenic *KMT2B* variant [8]. The remaining 16% of these reported affected patients have an inherited *KMT2B* pathogenic variant, with 10% attributable to an affected parent and an additional 6% attributable to a clinically asymptomatic parent. Within a family of *KMT2B* individuals, reduced penetrance and clinical variability have been hypothesized, consistent with other dominant genetic dystonias [9,10]. In this case report, we report a previously unidentified *KMT2B* pathogenic variant among three family members with varying clinical presentations ranging from mild developmental delay to progressively worsening dystonia, spasticity, and impaired speech.

## Case Presentation

Our proband was the product of an uncomplicated term pregnancy and delivery, with infantile developmental milestones reached appropriately. As a child, mild developmental delay was noted, and he received special education services. Dysmorphic features include mildly down-slanted palpebral fissures, slight rightward nasal deviation, elongated chin with retrognathia, and mild asymmetry of the lower back. At age 10, he developed dysarthria characterized as “froggy” with frequent voice breaks. Symptoms were exacerbated by stress and improved with whispering. Symptom severity fluctuated throughout the day without any temporal patterns identified. Vocal symptoms rapidly worsened at age 18. Video laryngoscopy revealed supraglottic hyperfunction consistent with laryngeal dystonia.

By age 19, our proband’s dystonia had progressed into unremitting difficulty walking with episodes of ascending lower extremity spasms and pain. Neurologic evaluation by a movement disorder specialist revealed slow saccades, lower extremity spasticity, subtle dystonic posturing of left upper extremity, and dystonic posturing of left leg. Additional findings included slightly stooped posture, decreased arm swing, everted gait, difficulty walking, and short stature (155 cm). Brain MRI was unremarkable. Attempted therapies included speech therapy, botulinum injections, baclofen, and carbidopa-levodopa.

Family history is notable for a number of family members with intellectual disabilities (ID). The proband’s sister experienced learning delays and received special education services. She also has a history of Attention Deficit Hyperactivity Disorder (ADHD), short stature, and subtle dystonia. The proband’s mother has a history of short stature, learning difficulties, and received special education. The maternal grandfather’s sister has a history of severe ID.

The proband’s father has a history of learning difficulties and benefitted from special education services. Remainder of paternal family history is significant for an uncle and great uncle with severe ID.

Despite an extensive family history of intellectual disabilities, no family members share a similar presentation to the proband. Specifically, there are no family members with dystonia, abnormal gait or dysphonia, with the exception of the proband’s sister who was identified to have subtle dystonia.

**Figure 1:**
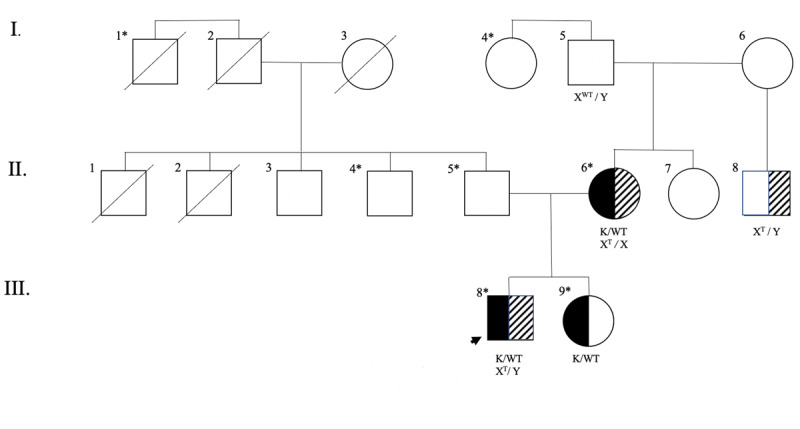
Family history of the proband. Black shading indicates heterozygosity of *KMT2B* c.5047 C > T (p.Q1683X). Diagonal striped shading indicates heterozyosity or hemizygsity of *TAF1*, c.3296 C > T (p.S1099L). WT–wildtype. K–*KMT2B* c.5047 C>T (pQ1683X). T–*TAF1*, c.3296 C>T (p.S1099L). * Indicates intellectual disability.

Family pedigree is displayed in ***Figure 1***.

### Genetic Test Results

Family members had not previously received genetic testing. Given the proband’s history of developmental delays, a chromosomal microarray was pursued initially and revealed a normal male result. Subsequent whole exome sequencing identified a maternally-inherited heterozygous pathogenic variant in *KMT2B*, c.5047 C>T (p.Q1683X). The *KMT2B* p.Q1683X variant is a nonsense variant, expected to cause early termination of protein translation or nonsense mediated decay. This variant has not been previously reported in the literature or ClinVar and is absent from population databases (gnomAD). Nonsense variants are a known mechanism for disease in *KMT2B* [2,3]. For these reasons, the variant was classified as Pathogenic, or disease-causing. Targeted testing also identified this *KMT2B* variant in the proband’s sister.

Additionally, there was a variant of uncertain significance (VUS) identified in X-linked *TAF1* (c.3296 C>T, p.S1099L) present in the proband and proband’s mother. The *TAF1* variant is a missense variant, with in-silico analysis supporting a deleterious effect on protein structure and function (-3.75 Provean). This variant has not been previously reported in the literature or ClinVar, and is absent from population databases (gnomAD). There are also no close residues reported in ClinVar or literature. Missense variants have been reported as disease-causing in the literature, however at different domains [15]. Exon 22, where this variant is located, is outside any domains known in *TAF1* [15]. Follow up family studies were performed to help clarify significance of the *TAF1* variant. Targeted *TAF1* testing on maternal male members demonstrated the same *TAF1* variant in an unaffected maternal half-uncle but not the unaffected maternal grandfather.

Mitochondrial genome sequencing with deletion analysis was negative.

## Discussion

Genetic testing of the proband revealed a pathogenic variant in *KMT2B* c.5047 C>T (p.Q1683X) and a VUS in *TAF1* (c.3296 C>T, p.S1099L). We believe that the *KMT2B* variant is responsible for the proband’s clinical manifestations, rather than *TAF1*. Genetic testing of additional family members supports this as the proband’s mother and sister carried the same *KMT2B* variant, but only the mother and unaffected maternal half-uncle possessed the *TAF1* variant. There is no known link between *TAF1* and *KMT2B*. In further assessing the likelihood of *TAF1* contributing to the proband’s presentation, *TAF1*-associated disorders were considered including X-linked dystonia-parkinsonism (XDP) and X-linked syndromic intellectual developmental disorder-33 (XSIDD-33) [15,16].

XDP is a progressive, highly penetrant disorder characterized by parkinsonism and rapidly progressive dystonia [16,17,18]. This disorder has been reported exclusively in those of Filipino descent with a specific retrotransposon insertion into *TAF1*. Our proband denies Filipino ancestry and his *TAF1* variant is distinctly separate from the XDP variant. XSIDD-33 is characterized by global delay, ID, dysmorphic facial features, joint hypermobility, and generalized hypotonia. Associated pathogenic *TAF1* variants ranged from deletions to missense mutations with all affected males exhibiting ID and global developmental delay. Other than developmental delay and dystonia, our proband lacks additional findings consistent with XSIDD-33. The proband’s maternal half-uncle possessed the same *TAF1* variant, but lacked any neurologic involvement, development delay, ID, or other findings consistent with a *TAF1*-related disorder. While variable expression and intrafamilial variability of *TAF1*-associated disorders has been reported, all males are expected to exhibit some phenotype due to their hemizygous state. Therefore, it is unlikely that *TAF1* was contributing to our patient’s constellation of findings.

The *KMT2B* variant in our proband is predicted to result in either a truncated protein or nonsense mediated decay and has not been previously observed in large population studies [11,12,13]. This variant is a stop-gain mutation, resulting in a shortened protein, suggesting that truncation at this point leads to loss of function.

Previous reports have noted stop-gain, splice site, and frameshift mutations and microdeletions in *KMT2B*-related dystonia patients resulted in earlier onset of focal dystonia (mean age 4.1 years) as compared to missense variants who developed dystonia later (mean 6.4 years of age) [2]. Additionally, those with *KMT2B*-dystonia have demonstrated reduced KMT2B expression. In our proband, despite a stop-gain *KMT2B* variant, he did not develop focal dystonia until age 10, supporting later range of symptom onset than previously reported. His disease progression was unique in that initial manifestation was isolated laryngeal dystonia for ten years, rather than limb dystonia. Similar to other *KMT2B*-dystonia patients, his condition progressed from focal to generalized dystonia and involved impaired ambulation, characteristic facial features, and intellectual disability.

Inherited variants in *KMT2B* with intrafamilial clinical variability has previously been reported on in at least two separate accounts [2,4]. In one family, the mother was reported to have a a milder presentation than the affected child [2]. In two additional families, clinically asymptomatic mothers carried the same *KMT2B* variant as affected offspring. Identification of symptomatic and asymptomatic carriers of the same *KMT2B* variant, supported by our proband and family, and previous literature, further supports the role of variable expressivity that may occur. Additionally, other environmental and epigenetic factors should also be considered in contributing to the variable phenotypes associated with a genotype. Further identification of *KMT2B* variants will allow for better appreciation of the phenotypic spectrum and disease progression.

## Abbreviations

KMT2B – Lysine methyltransferase 2B

MLL4 – Mixed Lineage Leukemia

H3K4 - histone H3 and lysine 4

TAF1 – TATA-box binding protein associated factor 1

ADHD - attention-deficit/hyperactivity disorder

VUS - Variant of unknown significance

ID - Intellectual disability
